# Prognostic significance of the red blood cell distribution width that maintain at high level following completion of first line therapy in mutiple myeloma patients

**DOI:** 10.18632/oncotarget.24076

**Published:** 2018-01-10

**Authors:** Yongyong Ma, Zhouxiang Jin, Shujuan Zhou, Haige Ye, Songfu Jiang, Kang Yu

**Affiliations:** ^1^ Department of Hematology, The First Affiliated Hospital of Wenzhou Medical University, Wenzhou 325000, China; ^2^ Department of Hepatobiliary Surgery, The Second Affiliated Hospital and Yuying Children’s Hospital of Wenzhou Medical University, Wenzhou 325027, China

**Keywords:** mutiple myeloma, red blood cell distribution width, first-line therapy, prognosis, survival

## Abstract

To investigate the prognostic value of the red blood cell distribution width(RDW) recovery from low levels at diagnosis after completion of first line therapy in mutiple myeloma (MM)patients,we enrolled 78 consecutive patients with MM and followed up from 2005 to 2016 in our hospital. The RDW was measured following completion of first-line therapy.The log-rank test, univariate analysis, and Cox regression analysis were used to evaluate the relationship between RDW and survival. We found that patients with an RDW ≥ 15.5% at diagnosis, as well as at completion of first-line therapy, had significantly lower progression-free survival (PFS) and overall survival(OS) rates than those with an RDW < 15.5%(*P* < 0.05).Patients with RDW that maintained more than 15.5% upon completion of therapy showed a shorter OS (*P* < 0.05) and PFS (*P* < 0.05) compared with patients with an RDW that decreased to a lower level.The multivariate analysis showed that RDW ≥ 15.5% after the completion of first-line therapy were an independent prognostic marker of poorer OS (*P* = 0.044) and PFS (*P* = 0.034). Therefore,we demonstrated that RDW at diagnosis, as well as at completion of first-line therapy is an independent predictor for mutiple myeloma patients.RDW maintained at high level, irrespective of whether RDW decreased to the cutoff value predicted an unfavorable prognosis in patients with MM.

## INTRODUCTION

Multiple myeloma (MM) is an incurable blood cancer of plasma cells characterized by the clonal proliferation of plasma cells in the bone marrow and production of monoclonal proteins in the blood or urine. Patients with MM have highly variable prognoses [1]. The International Staging System (ISS) and DurieSalmon staging system (DSS) are the most widely accepted prognostic scoring system for patients with MM, however, some patients with a favorable ISS or DSS fail treatment and vice versa. Prognostic factors are required to distinguish low risk disease from aggressive forms.But MM has a heterogeneous spectrum of clinical entities and outcome. Due to wide variation in outcome there is need to define other prognostic variables [2].

Several molecular markers of MM have been identified, but cost and technical limitations make their routine application impractical [3–4].Hence the search for a simple, inexpensive, routinely measured prognostic biomarker will be of great importance for MM patients. Red blood cell distribution width (RDW) is a simple, inexpensive, routinely measured and automatically reported blood test parameter, which reflects the degree of anisocytosis of red blood cells in peripheral blood [5–7]. Previous studies in literature have shown that red blood cell distribution width are important prognostic marker in various solid cancers and hematology tumors [8–16]. RDW and its correlation with other prognostic factors can play an important role in risk stratification of MM patients [17–19].

Recent studies have shown that the RDW obtained at diagnosis using the complete blood count (CBC) may predict clinical outcomes in MM,the overall response rates (ORR) and complete response (CR) rate of initial therapy were markedly higher in the low-RDW group compared to the high-RDW group. RDW was significant lower in CR in comparison to Non-CR groups in patients treated with bortezomib-based regimens as induction therapy [18–19]. The patients with low-RDW at diagnosis had better OS when compared to those with high-RDW.However, the effects of RDW maintained at high level upon diagnosis, after therapy, remain unclear. Thus, we explored whether peripheral RDW maintained at high level at diagnosis, after completion of first-line therapy, can predict clinical outcomes in MM.

## RESULTS

### Patient population

There were 78 patients enrolled in this retrospective study [median age = 60.7 years (range: 43–81 years); 47 (60.2%) males].The male to female ratio was 1.51:1. The majority of MM subtype was IgG (47.4%).IgA account for 25.6% MM patients and 23.1% of 78 MM patients had light chain disease.According to the ISS,5 patients were stage I (6.4%), 46 patients stage II (59.0%) and 27 patients stage III (34.6%).Regarding the DS,5 patients were stage I (6.4%), 19 patients stage II (24.4%) and 54 patients stage III (69.2%).The distribution of additional baseline characteristics of these patients are shown in Table 1. Median follow-up after diagnosis was 42.6 months for the entire cohort (range 2 to 136 months) and for censored observations. A total of 57 patients experienced relapse, disease progression, or death. The median PFS was 33.1 months (range 1–113 months), while the median OS was 42.6 months (range 2–136 months).

**Table 1:** Baseline patients’ characteristics based on RWD at diagnosis and at completion of therapy

Characteristics	Total (*n* = 78)	at diagnosis(%)			RWD at completion of therapy (%)		
		> 15.5 (*n* = 46)	< 15.5 (*n* = 32)	*P*-value	> 15.5 (*n* = 35)	< 15.5 (*n* = 43)	*P*-value
Sex, male	48(60.2%)	30(65.2%)	17(53.1%)	0.283	20(57.1%)	27(62.8%)	0.612
Age > 60 years	37(47.4%)	22(47.8%)	15(46.9%)	0.934	18(51.4%)	19(44.2%)	0.524
ECOG PS > 2	3(3.8%)	1(2.2%)	2(6.3%)	0.357	1(2.9%)	2(4.7%)	0.682
ISS stage				0.157			0.672
I/II	51(65.4%)	33(71.7%)	18(56.3%)		22(62.9%)	29(67.4%)	
III	27(34.6%)	13(28.3%)	14(43.8%)		13(37.1%)	14(32.6%)	
DS stage				0.283			0.544
I/II	24(30.8%)	12(26.1%)	12(37.6%)		12(34.3%)	12(27.9%)	
III	54(69.2%)	34(73.9%)	20(62.5%)		23(65.7%)	31(72.1%)	
Isotype							
IgG, κ, or λ	37(47.4%)	22(47.8%)	15(46.9%)	0.934	19(54.3%)	18(41.9%)	0.274
IgA, κ, or λ	20(25.6%)	10(21.7%)	10(31.3%)	0.344	9(25.7%)	11(25.6%)	0.141
Light chain disease	18(23.1%)	12(26.1%)	6(18.8%)	0.449	7(20.0%)	11(25.6%)	0.561
others	3(3.8%)	2(4.3%)	1(3.1%)	0.782	0(0.00%)	3(7%)	-
Hemoglobin < 100 g/L	49(62.8%)	30(65.2%)	19(59.4%)	0.599	20(57.1%)	29(67.4%)	0.349
Creatinine > 176.8 μmol/L	12(15.4%)	9(19.6%)	3(9.4%)	0.220	6(17.1%)	6(14%)	0.698
Calcium > 2.75 mmol/L	9(11.5%)	5(10.9%)	4(12.5%)	0.825	2(5.7%)	7(16.3%)	0.146
Albumin < 35 g/L	45(57.7%)	27(58.7%)	18(56.3%)	0.830	20(57.1%)	25(58.1%)	0.929
β2-microglobulin > 5.5 mg/L	36(46.2%)	20(43.5%)	16(50.0%)	0.570	15(42.9%)	21(48.8%)	0.598
BM plasma cell ≥ 30%	35(44.9%)	20(43.5%)	15(46.8%)	0.767	17(48.6%)	18(41.9%)	0.553
osteolytic bone lesions ≥ 3	40(51.3%)	21(45.7%)	19(59.4%)	0.233	17(48.6%)	23(53.5%)	0.666
Cytogenetics (FISH)							
1q21 amplification	38(48.7%)	18(39.1%)	20(62.5%)	0.042	17(48.6%)	21(48.8%)	0.981
13q14 deletion	14(17.9%)	6(13.0%)	8(25.0%)	0.176	5(14.3%)	9(20.9%)	0.447
p53 deletion	15(19.2%)	7(15.2%)	8(25.0%)	0.281	8(22.9%)	7(16.3%)	0.463
IgH rearrangement	53(67.9%)	32(69.6%)	21(65.6%)	0.714	25(71.4%)	28(65.1%)	0.552
Front-line treatment							
bortezomib-based regimen	78(100%)	46(100%)	32(100%)	-	35(100%)	43(100%)	-
thalidomide-based regimen	17(21.8%)	11(23.9%)	6(16.7%)	0.587	6(17.1%)	11(25.6%)	0.369
VAD regimen	2(2.6%)	1(2.2%)	1(3.11%)	0.794	1(2.9%)	1(2.3%)	0.883
SCT	12(15.4%)						
Auto	9(11.5%)	7(15.2%)	2(6.3%)	0.223	3(8.6%)	6(14.0%)	0.459
Sibling-matched	3(3.8%)	1(2.2%)	2(6.3%)	0.357	1(2.9%)	2(4.7%)	0.682

RWD, red blood cell distribution width; ECOG, Eastern Cooperative Oncology Group; PS, performance status; ISS, international staging system; LDH, lactate dehydrogenase; ULN, upper limit of normal value;FISH, fluorescent in situ hybridization; BM, bone marrow; SCT, stem cell transplantation.

### RDW at diagnosis and upon completion of chemotherapy and clinical outcomes

**Figure 1:**
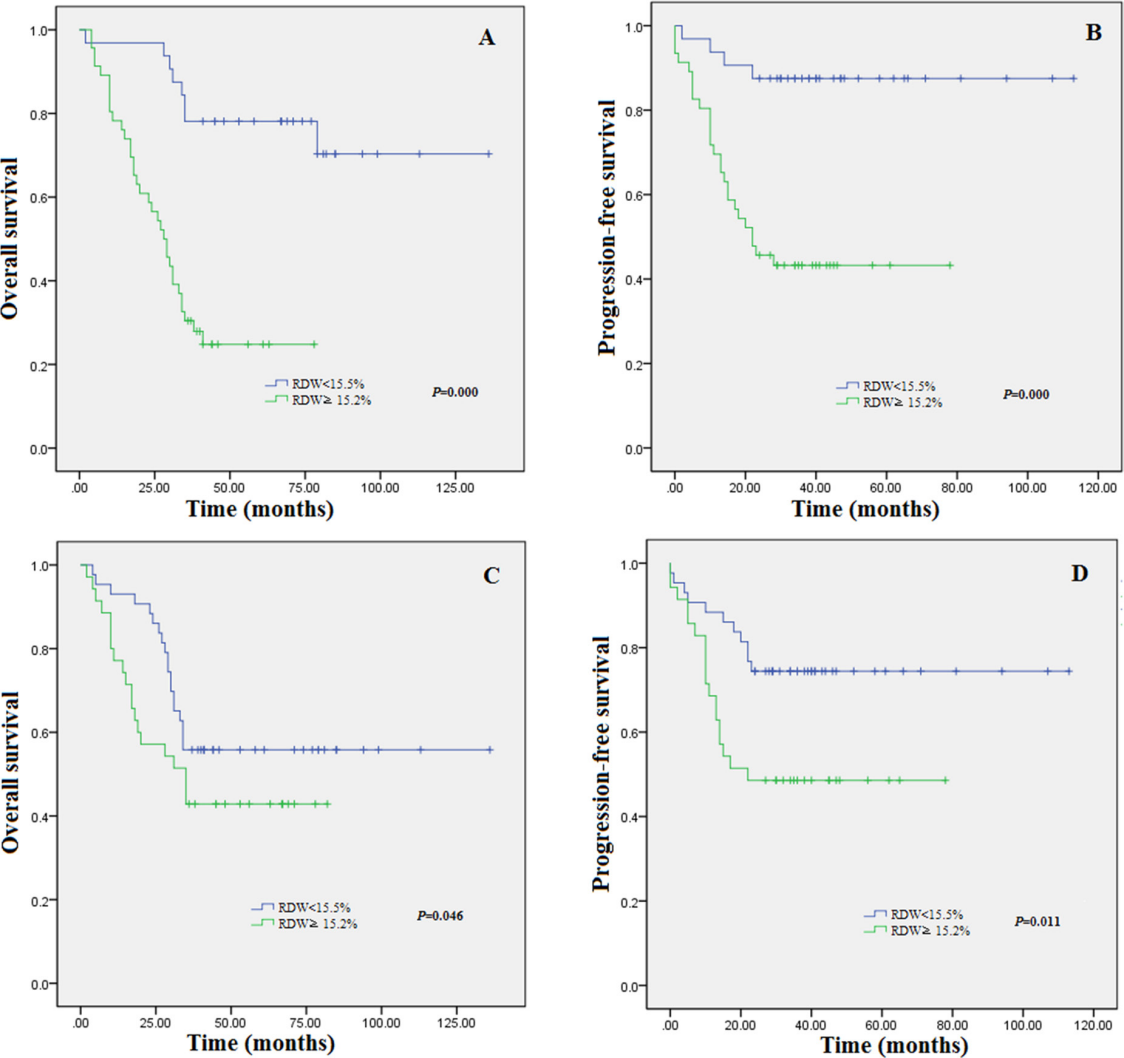
Kaplan-Meier estimates of overall survival (**A**) and progression-free survival (**B**) for the 78 MM patients stratified by RDW at diagnosis. Kaplan-Meier estimates of overall survival (**C**) and progression-free survival (**D**) for the 78 MM patients stratified by RDW at the completion of therapy.

The median RDW at diagnosis was 15.46% (range:12.0%–18.4%). A total of 46 (59.0%) patients had RDW ≥ 15.5% and 32 (41.0%) had RDW < 15.5% at diagnosis. RDW ≥ 15.5% at diagnosis was significantly correlated with 1q21 amplification (*P* = 0.042).As shown in Figure 1, patients with a low RDW < 15.5% had significantly higher OS rate [Figure 1(A), *P* = 0.000] and PFS Figure 1B, *P* = 0.000] than those with an RDW ≥ 15.5% at diagnosis. The median RDW upon completion of therapy was 14.9% (range:12.1–18.8%). 33(42.3%) patients decreased to a lower RDW at the completion of therapy, but 12(15.4%) patients failed to do so. A total of 43 (55.1%) had RDW ≥ 15.5% and 35 patients (44.9%) had RDW < 15.5% upon completion of therapy.The patients’ baseline of RDW ≥ 15.5% and RDW < 15.5% upon completion of therapy was similar. However,as shown in Figure 1, patients with an RDW < 15.5% had significantly higher OS [Figure 1C, *P* = 0.046] and PFS [Figure 1D, *P* = 0.011] than those with an RDW ≥ 15.5% upon completion of therapy.We found that patients whose RDW ≥ 15.5% upon completion of therapy experienced poorer prognosis. Therefore,we investigated whether patients who started with a high RDW ≥ 15.5% at diagnosis but then obtained a low RDW < 15.5% showed longer survival compared to patients maintained at high RDW ≥ 15.5% at the completion of therapy.In addition,we explored whether patients who started with a low RDW < 15.5% at diagnosis but then obtained a high RDW ≥ 15.5% upon completion of therapy showed shorter survival compared with patients who maintained a low RDW < 15.5% upon completion of therapy.

**Figure 2:**
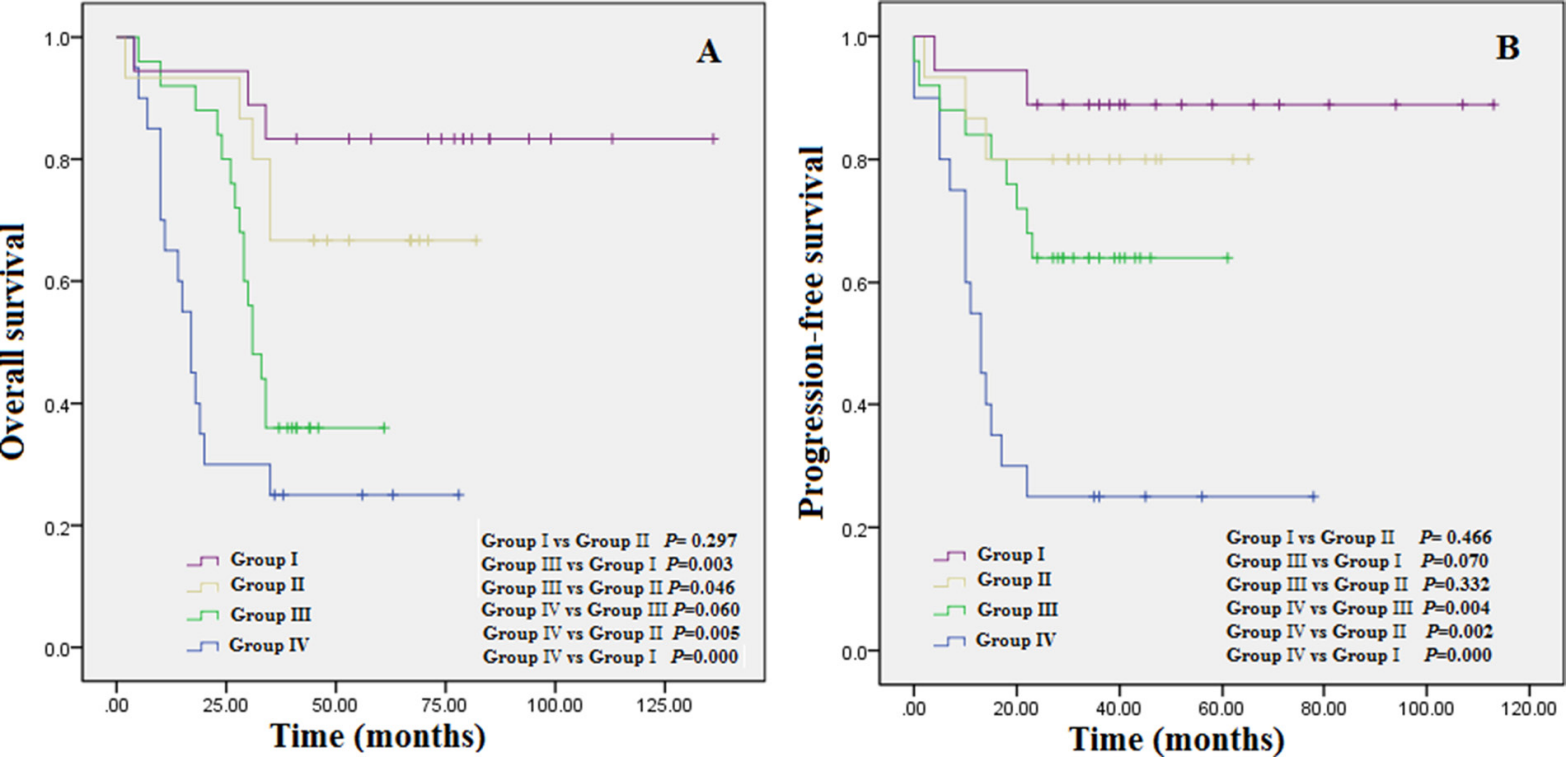
(**A**) Overall survival based on group stratification; (**B**) progression-free survival based on group stratification: Group I = patients with an RDW < 15.5% at diagnosis and at the completion of therapy; group II = patients with an RDW < 15.5% at diagnosis but then obtained an RDW ≥ 15.5% at the completion of therapy; group III = patients with a high RDW ≥ 15.5% at diagnosis but then gained an RDW < 15.5% at the completion of therapy; and group IV = patients with a high RDW ≥ 15.5% at diagnosis and at the completion of therapy.

To figure out these questions, patients were divided into four subgroups. Group I consisted of patients with an RDW < 15.5% at diagnosis and at the completion of therapy; group II included patients with an RDW < 15.5% at diagnosis but then obtained an RDW ≥ 15.5% at the completion of therapy; group III included patients with a high RDW ≥ 15.5% at diagnosis but then gained an RDW < 15.5% at the completion of therapy; and group IV included patients with a high RDW ≥ 15.5% at diagnosis and at the completion of therapy. As desired, grounded on cluster analysis, Although patients in group I obtained longer OS and PFS compared to the other groups Figures 2A and 2B, and patients in group IV experienced shorter OS and PFS compared to the other groups Figures 2A and 2B, group IV experienced shorter OS and PFS compared to group III, suggesting that RDW ≥ 15.5% upon completion of therapy resulted in an poorer clinical outcome and also implying that an RDW < 15.5% irrespective of an RDW ≥ 15.5% at diagnosis was related with improved clinical outcomes.

**Figure 3:**
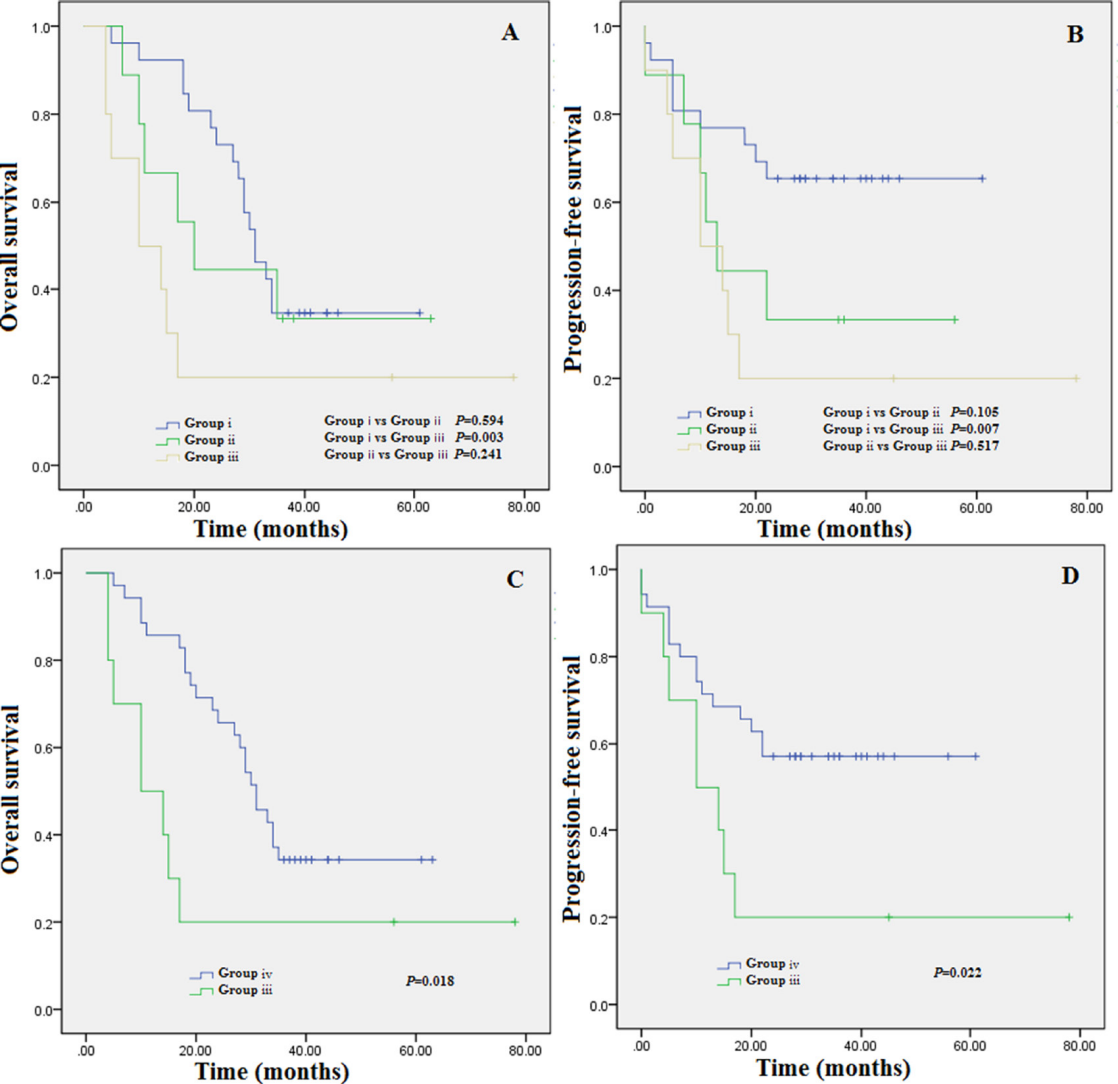
(**A**) Overall survival based on group stratification; (**B**) progression-free survival based on group stratification; (**C**) Overall survival based on group stratification,group iii vs group i+ii;D.progression-free survival based on group stratification,group iii vs group i+ii.Group i = patients with an RDW < 15.5% upon completion of therapy; group ii = patients with an RDW decreased to a lower value, but the RDW value maintained more than 15.5% upon completion of therapy; group iii = patients who then failed to decrease to a lower value and maintain a high level RDW ≥ 15.5% upon completion of therapy. Group i plus group ii makes group iv.

Patients with RDW ≥ 15.5% at diagnosis but RDW< 15.5% upon completion of therapy showed longer OS and PFS. We then observed that compared with the patients with a high RDW ≥ 15.5% upon completion of therapy,patients who started with a high RDW ≥ 15.5% at diagnosis and then decreased to a lower value, but the value did not lower than 15.5% upon completion of therapy, presented longer survival.To elucidate the problems, patients who started with a high RDW ≥ 15.5% at diagnosis were classified into four subgroups. Group i consisted of patients with an RDW < 15.5% upon completion of therapy.Group ii obtained patients with an RDW decreased to a lower value, but the RDW value maintained more than 15.5% upon completion of therapy. Group iii obtained patients who then failed to decrease to a lower value and maintain a high level RDW ≥ 15.2%upon completion of therapy.Group i plus group ii makes group iv. As desired from the cluster analysis, patients in group i showed longer OS and PFS compared to the group iii Figures 3A and 3B. Although we found there as no statistically significant enhanced OS (*P* = 0.241) and PFS (*P* = 0.517), group ii obtained longer OS and PFS compared to group iii, implying that RDW approaching to 15.5% upon completion of therapy even if the MM patients started with a high RDW ≥ 15.5% at diagnosis led to a superior clinical outcome.Based on the cluster analysis showing that patients with a high RDW, who did not decrease a lower RDW value upon completion of therapy, obtained the most poor clinical outcomes, group iv obtained longer OS and PFS compared to group iii. The distribution of additional baseline characteristics of group iii and group iv patients are shown in Table 2. Using Kaplan-Meier curves, patients maintained at high RDW value experienced shorter OS Figure 3C, *P* = 0.018] and PFS Figure 3D, *P* = 0.022 compared with patients with a low RDW upon completion of therapy.

**Table 2:** Baseline patients’ characteristics with a high RDW ≥ 15.5% at diagnosis based on RDW decreased versus RDW maintain at high level following completion of first line therapy

Characteristic (*n* = 46)	RDW decrease		*P*
	Yes (*n* = 34 group iv)	No (*n* = 12 group iii)	
Sex, male	20 (58.8%)	10 (83.3%)	0.125
Age > 60 years	16 (47.1%)	6 (50.0%)	0.861
ECOG PS > 2	1 (2.9%)	0 (0%)	-
ISS stage			0.23
I/II	26 (76.5%)	7 (58.3%)	
III	8 (23.5%)	5 (41.7%)	
DS stage			0.387
I/II	10 (29.4%)	2 (16.7%)	
III	24 (70.6%)	10 (%)	
Isotype			
IgG, κ, or λ	18 (52.9%)	4 (33.3%)	0.242
IgA, κ, or λ	6 (17.6%)	4 (33.3%)	0.257
Light chain disease	9 (26.5%)	3 (25.0%)	0.921
others	1 (2.9%)	1 (8.3%)	0.431
Hemoglobin < 100 g/L	22 (64.7%)	8 (66.7%)	0.902
Creatinine > 176.8 μmol/L	5 (14.7%)	4 (33.3%)	0.162
lcium > 2.75 mmol/L	3 (8.82%)	2 (16.7%)	0.453
Albumin < 35 g/L	19 (55.9%)	8 (66.7%)	0.514
β2-microglobulin > 5.5 mg/L	14 (41.2%)	6 (50.0%)	0.596
BM plasma cell ≥ 30%	14 (41.2%)	6 (50.0%)	0.514
osteolytic bone lesions ≥ 3	15 (44.1%)	6 (50.0%)	0.725
Cytogenetics (FISH)			
1q21 amplification	14 (41.2%)	4 (33.3%)	0.632
13q14 deletion	5 (14.7%)	1 (8.3%)	0.573
P53 deletion	5 (14.7%)	2 (16.7%)	0.871
IgH rearrangement	24 (70.6%)	8 (66.7%)	0.800
Front-line treatment			
bortezomib-based regimen	34 (100.0%)	12 (%)	-
thalidomide-based regimen	7 (20.6%)	4 (33.3%)	0.374
VAD regimen	1 (2.9%)	0 (0.00%)	-
SCT	6 (17.6%)	2 (16.7%)	0.939

### Univariate and multivariate analyses

Results of the univariate and multivariate analysis for factors influencing OS and PFS in patients with MM are reported in Table 3. Univariate Cox-regression analysis showed that prognostic factors for OS were with ISS stage (*P* = 0.034), RDW ≥ 15.5% after treatment (*P* = 0.012) ,RDW maintain at high level after treatment (*P* = 0.034)and β2-microglobulin > 5.5 mg/L (*P* = 0.012). Multivariate analysis that included all the parameters having a *P* value of less than 0.05 in the univariate analysis revealed that ISS stage (*P* = 0.034), RDW ≥ 15.5% after treatment (*P* = 0.044) and β2-microglobulin > 5.5 mg/L (*P* = 0.018) are independent prognostic factors for OS.Univariate Cox-regression analysis indicated that prognostic factors for PFS were with ISS stage (*P* = 0.005),and RDW ≥ 15.5% after treatment (*P* = 0.037).Multivariate analysis revealed that ISS stage (*P* = 0.005) and RDW ≥ 15.5% after treatment (*P* = 0.034) are independent prognostic factors for PFS.

**Table 3:** Univariate and multivariate analyses for analysis for overall survival and progression-free survival in patients with a high RDW ≥ 15.5% at diagnosis

Variable	Overall survival			Progression free survival		
	Univariate analyses *p*-value	HR (95% CI)	Multivariate analyses *p*-value	Univariate analyses *p*-value	HR (95% CI)	Multivariate analyses p-value
Sex, male	0.098			0.106		
Age > 60 years	0.818			0.737		
ECOG PS > 2	1.000			1.000		
ISS stage	0.034	0.208 (0.049–0.886)	0.034	0.005	0.111 (0.024-0.520)	0.005
DS stage	0.401			0.857		
Hemoglobin < 100 g/L	0.313			0.829		
Creatinine > 176.8 μmol/L	0.655			0.821		
Calcium > 2.75 mmol/L	0.999			0.358		
Albumin < 35 g/L	0.282			0.254		
β2-microglobulin > 5.5 mg/L	0.012	22.364 (1.689–296.189)	0.018	0.370		
BM plasma cell ≥ 30%	0.329			1.000		
osteolytic bone lesions ≥ 3	0.500			0.950		
SCT	0.145			0.107		
RDW ≥ 15.5% after treatment	0.012	5.263 (1.055–7.462)	0.044	0.037	3.891 (1.083-13.889)	0.034
RDW maintain at high level after treatment	0.034	1.308 (0.162–10.526)	0.800	0.397		

## DISCUSSION

RDW is a measure of size variability[8] and heterogeneity of erythrocytes in the peripheral blood (i.e., anisocytosis) and is routinely measured in clinical practice as part of a complete blood count (CBC). More recent studies have investigated the role of RDW in patients with various cancer [9–11], and RDW was proved to be an independent prognosis factor in prostate cancer, chronic lymphocytic leukemia,esophageal cancer, hepatocellular carcinoma,breast cancer, diffuse large B-cell lymphoma,malignant mesothelioma,upper tract urothelial Carcinoma,myeloma and so on [8–16].We evaluated the OS and PFS by comparing RDW upon completion of therapy to the baseline at diagnosis. We found that patients whose RDW ≥ 15.5% upon completion of therapy experienced poorer prognosis. In addition,we found patients who started with a high RDW ≥ 15.5% at diagnosis but then obtained a low RDW < 15.5% showed longer survival compared to patients maintained at high RDW ≥ 15.5% at the completion of therapy. Furthermore,that patients with a high RDW, who did not decrease to a lower RDW value upon completion of therapy, obtained the most poor clinical outcomes.

Our results are in accord with the study by Wang et al [17–18], who observed patients with multiple myeloma and found that high RDW levels had a significantly high correlation with some unfavorable clinical parameters and cytogenetic abnormalities and an elevated RDW value was independently associated with a poor PFS in MM patients. However, there are still several important questions to explore.The first question involves the cutoff values of the RDW at diagnosis, which is a threshold value for a quantity,predicting clinical outcomes in patients with MM, is needed in practical clinical work.In our study,the optimal threshold is 15.5%.Moreover,to avoid the cut-off affected by the incidence and patient characteristics,we also compared the RDW upon completion of chemotherapy to the baseline,in patients with higher RDW at diagnosis. In addition, we did find it had prognostic significance. The second problem relates to the mechanism that MM with increased RDW levels,which involve shortening of telomere length, oxidative stress,inflammation,deregulation of iron metabolism,poor nutritional status (i.e., deficiencies in nutrients such as iron, vitamin B12, and folate),dyslipidemia, hypertension, erythrocyte fragmentation,inadequate production and alteration of erythropoietin function,erythrocyte maturation impairment,changes in red blood cell maturation by altering the red cell membrane and the impaired iron release from reticuloendothelial macrophages [20–23]. The possible explain is that the low RDW represents the normal metabolic status, and RDW < 15.5% represents the normal RDW range. Thus, patients with low RDW at diagnosis have no correlation with whether RDW increased after the completion of therapy, as long as the RDW remained at low levels. While the high RDW ≥ 15.5% represents host metabolic abnormalities.

Therefore,decreased RDW after therapy is representative of metabolic status recovery. Which also means the patients benefit from chemotherpy and they had a better prognosis.In a similar way,RDW remain at high level at diagnosis, after the completion of therapy, is associated with poor clinical outcomes.

One of the limitations of the present study was the retrospective nature, recruitment was performed in a single institution. further multicenter prospective studies are required.Another potentially limiting factor of our study is that the translocation of IgH was detected by an IgH break-apart rearrangement probe but not by t(4;14)and t(14;16) probes due to the high fee. Furthermore,we had no complete and detailed information on the influence factors for RDW in MM, such as Interleukin 6 and serum ferritin levels for the total cohort, thus we could not adjust the association of RDW with risk of death for these influence factors [24–26].

In conclusion, the results in this study indicated that RDW at diagnosis, as well as at completion of first-line therapy is an independent predictor for mutiple myeloma patients.RDW maintained at high level, irrespective of whether RDW decreased to the cutoff value predicted an unfavorable prognosis in patients with MM.RDW which is a part of complete blood count and is a routinely measured and automatically generated blood paramete,may represent one of the easiest measurements to be used as a prognostic marker in patients with MM. Further investigations are required to illustrate the exact mechanisms underlying the influence of distribution width on red blood cells in patients with MM.

## MATERIALS AND METHODS

### Patients and methods

The inclusion criteria were a diagnosis of de novo MM according to the criteria of NCCN guideline,and all patients were treated at the First Affiliated Hospital of Wenzhou Medical University between February 2005 and December 2016.Patients who had the complete information and details concerning laboratory parameters were included.Patients who were diagnosed with monoclonal gammopathy of undetermined significance, asymptomatic MM, amyloidosis, and plasma cell leukemia were excluded.Written informed consent was obtained from each patient before entering the study according to the Declaration of Helsinki, and the study was approved by the Institutional ethics committee.

Bortezomib-based regimen were used as first-line therapy in all 78 patients,which included PAD (bortezomib, adriamycin and dexamethasone), VD(bortezomib and dexamethasone) and PCD (bortezomib,cyclophosphamide and dexamethasone).

Thalidomide-based regimen which included TD (thalidomide and dexamethasone), TCD (thalidomide, cyclophosphamide and dexamethasone) and MPT (melphalan, prednisolone and thalidomide) was used in 17 patients (21.8%).Two patients (2.6%) received VAD (vincristine, adriamycin and dexamethasone)as first-line chemotherapy.

In addition, 9 patients (11.5%) underwent auto-SCT and 3 (3.8%) received related allogeneic SCT.

### Laboratory data

RDW were obtained from standard complete blood cell count (CBC) data; The RDW upon completion of first-line therapy was calculated when the CBC reached a plateau after the bone marrow had recovered from first-line therapy. It is the standard practice of our clinicians to obtain a CBC 3 months after completion of chemotherapy. Therefore, we used RDW data from the 3-month follow-up visits.

**Figure 4:**
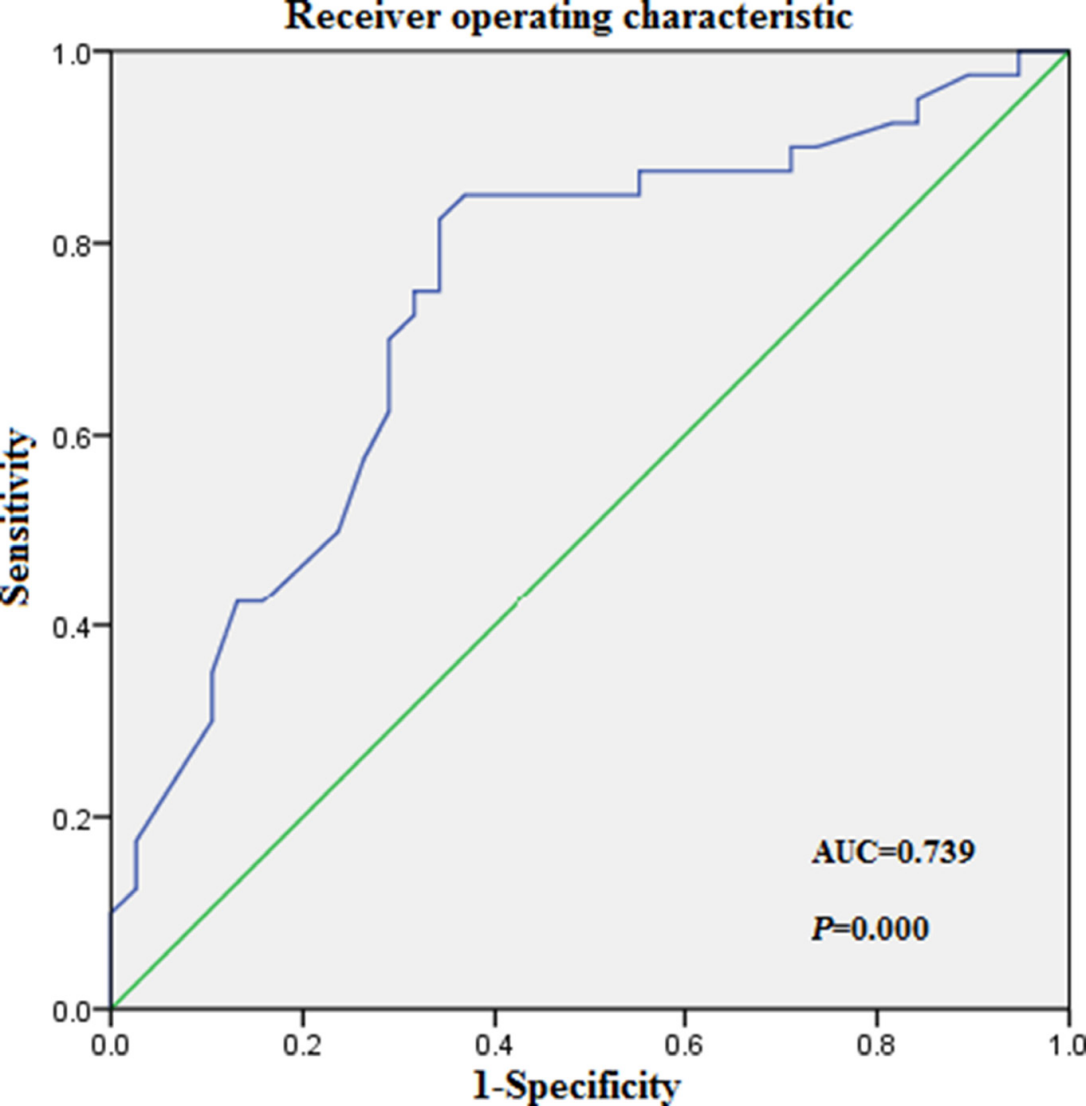
Receiver operating characteristic (ROC) curves analysis for RDW at diagnosis

The threshold of 15.5% was established as the maximum (sensitivity+specificity) point according to the area under curve (AUC) of the receiver operating characteristics curve (ROC; Figure 4). The binary clinical outcome (death/survival) was determined 3 years after diagnosis. Patients were categorized as “alive/censored” when the follow-up time was longer than 3 years and “dead” when they died before this time. Patients were further divided into two groups: low RDW group (RDW < 15.5%) and high RDW group (RDW ≥ 15.5%).

### Statistical analysis

The statistical analyses were performed using SPSS software (ver. 20.0).Correlations of the RWD with clinical parameters were evaluated using the chi-square or Fisher’s exact test. OS and PFS were analyzed using Kaplan-Meier curves, which were compared using the log-rank test. Receiver operating characteristic (ROC) curve analysis was used to determine the optimal RWD cutoffs yielding the maximal combined sensitivities and specificities. A *P* value of < 0.05 was considered statistically significant. Variables that were significant at *P* < 0.05 in the univariate Cox regression analysis were included in the multivariate analysis using forward stepwise selection.
